# Commentary: Acupuncture combined with balloon dilation for post-stroke cricopharyngeal achalasia: a meta-analysis of randomized controlled trials

**DOI:** 10.3389/fnins.2023.1214834

**Published:** 2023-08-24

**Authors:** Hua Jiang, Hongtao Zhang, Xinghua Zhang, Yanlong Xu

**Affiliations:** Department of Acupuncture and Moxibustion, Gansu Provincial Hospital of Traditional Chinese Medicine, Lanzhou, Gansu, China

**Keywords:** acupuncture, balloon dilation, stroke, cricopharyngeal achalasia, meta-analysis, commentary

We recently read an article in Frontiers in Neuroscience by Luo et al. entitled “*Acupuncture combined with balloon dilation for post-stroke cricopharyngeal achalasia: A meta-analysis of randomized controlled trials*” (Luo et al., 2023). The authors performed a meta-analysis and concluded that acupuncture combined with balloon dilatation may be an effective treatment for post-stroke cricopharyngeal achalasia. Notably, the authors explored the factors influencing this combination treatment from different perspectives through three subgroup analyses. However, we would like to further improve this important study from two aspects.

The authors highlight the use of the Cochrane Collaboration's risk-of-bias tool by two reviewers to independently assess the methodological quality of each trial. Our main concern was the lack of accuracy in the assessment of the risk-of-bias in the included trials. For example, in several studies (Zhang, 2017, 2019; Cao et al., 2019; Fan et al., 2020), the investigators did not report the method of randomization used, and, therefore, the risk of randomization should have been unknown, rather than low as described by the authors. In the study by Zhang (2017), the Methods section was too brief and did not present specific operational details of the observation and control groups or indicate specific outcome measures. Therefore, there was a high risk of selective publication bias and other biases. Therefore, we reassessed the methodological quality of all articles and presented them in Figure 1.

**Figure 1:**
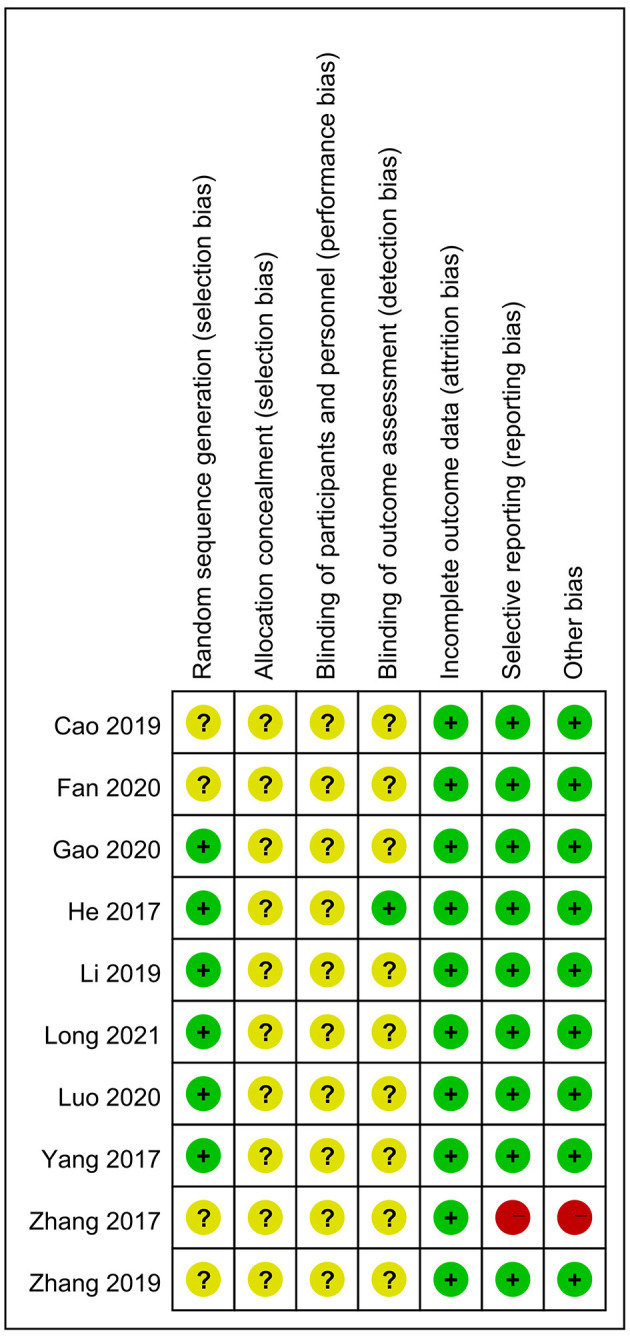
Risk-of-bias summary.

The included studies used two methods to assess treatment efficacy: the water swallow test (WST) and the videofluoroscopic swallowing study (VFSS). However, the authors only evaluated the difference between the two groups in the VFSS but did not evaluate the WST. Therefore, based on the WST data from three studies (Yang and Lei, 2017; Cao et al., 2019; Long et al., 2021), we found that the intervention group performed better on the WST (Figure 2).

**Figure 2:**
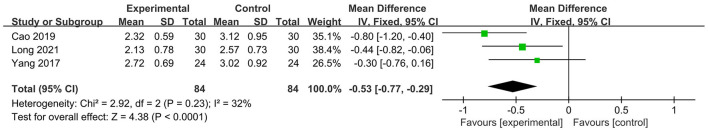
Forest plot for the WST score.

Despite these drawbacks, the benefits of balloon dilatation combined with acupuncture on swallowing function in stroke patients still deserve recognition. The results of this meta-analysis provide solid evidence for the inclusion of this combined intervention in a wide range of stroke patients with dysphagia. In future clinical practice, researchers will need to standardize the best of acupuncture to monitor long-term clinical outcomes. On the other hand, given the homogeneity of the study population (limited to Chinese patients), future studies should continue to expand to other national and regional populations.

## Author contributions

HJ wrote the paper. HZ drew the tables. XZ drew the graphs. YX reviewed the article.
